# Interleukin‐13 contributes to the occurrence of oral submucosal fibrosis

**DOI:** 10.1111/jcmm.17761

**Published:** 2023-06-19

**Authors:** Liping Wang, Zhangui Tang, Junhui Huang

**Affiliations:** ^1^ Hunan Key Laboratory of Oral Health Research & Hunan 3D Printing Engineering Research Center of Oral Care & Academician Workstation for Oral‐maxilofacial and Regenerative Medicine & Hunan Clinical Research Center of Oral Major Diseases and Oral Health & Xiangya Stomatological Hospital & Xiangya School of Stomatology Central South University Changsha China

**Keywords:** arecoline, IL‐13, M2‐macrophages, oral submucous fibrosis, polarization

## Abstract

Oral submucous fibrosis (OSF) is a chronic progressive fibrosis disease that affects in oral mucosal tissues. Interleukin (IL)‐13 has been implicated in the development of fibrosis in multiple organs. Indeed, it contributes to diseases such as pulmonary fibrosis, liver cirrhosis among others. Currently, its expression in OSF and the specific mechanisms are not well understood. The aim of this study was to investigate the role of IL‐13 in OSF and further explore whether IL‐13 regulates—polarization of M2‐macrophages in OSF. Initially, in the tissues of patients with OSF, we observed a high expression of M2‐macrophages and IL‐13 protein. Additionally, we found a correlation between the expression of IL‐13 and the stage of OSF. Arecoline inhibited the proliferation of fibroblasts (FBs) and promoted IL‐13 production in vitro. Furthermore, our observations revealed that M2‐macrophages increased upon co‐culturing M0‐macrophages with supernatants containing the IL‐13 cytokine. In conclusion, our study demonstrated that arecoline stimulates FBs leading to increased secretion of IL‐13, which in turn IL‐13 leads to polarization of M2‐macrophages and promotes the occurrence of OSF. This suggests that IL‐13 may be a potential therapeutic target of OSF.

## 1 INTRODUCTION

Oral submucous fibrosis (OSF) is a chronic progressive disease that primarily affects the oral mucosal tissues and has a predisposition towards malignancy. This disease, which was first described by Paymaster to be potentially malignant,^1^ has been classified as a precancerous condition by the World Health Organization (WHO). OSF prevalence was reported to be 1.00%–3.03% in Hunan Province,^2^ 0.15%–14.40% in Vietnam^3^ and 0.09%–17.60% in Taiwan.^4^ Despite being a potentially malignant condition, the precise pathogenesis of OSF remains unclear. Therefore, unravelling the pathogenesis and development of precise and efficient interventions is essential to the clinical management of the disease.

Studies have shown that the immune microenvironment of OSF significantly changes during OSF occurrence, including infiltration of immune cells of different subtypes and functions in the diseased tissue, as well as expression of immune‐related cytokines.^5^ For example, Chiang et al. found significant immune cell infiltration in tissues obtained from OSF patients, and macrophages were the main active cells. Macrophages can undergo various polarization pathways that results in significant functional alterations in the neighbouring cells and the extracellular matrix, and these directions can determine whether the microenvironment develops persistent inflammation (M1), or fibrosis (M2) after regeneration and/or injury.^6^, ^7^ Studies have demonstrated that M2‐macrophages directly affect development of fibrosis by producing a large number of pro‐fibrotic factors, such as transforming growth factor‐β1 (TGF‐β1), among others, thereby promoting proliferation of myofibroblasts, and overproducing the extracellular matrix.^8^, ^9^

Interleukins (ILs) are cytokines that play an important role in the activation, differentiation, proliferation, maturation and migration of immune cells.^10^ Studies have shown that IL‐13 contributes to the pathogenesis of fibroproliferative diseases, such as pulmonary fibrosis, liver cirrhosis, myocardial fibrosis, progressive kidney disease and pathological skin scarring, among others.^11^, ^12^, ^13^, ^14^ Furthermore, IL‐13 was associated with elevated production of total collagens in normal human skin and keloid fibroblasts (FBs), whereas FBs in various scarring diseases produced more fibrin, suggesting that IL‐13 is associated with fibrosis.^15^, ^16^, ^17^ Interestingly, a recent report demonstrated that in fibrosis, inhibition of both IL‐13 and TGF‐β signalling completely attenuated the fibrotic mechanism compared to TGF‐β inhibition alone.^18^

The Areca nut ranks fourth among the most commonly used addictive substances globally.^19^ Some scholars believe that several components in the areca nut, especially arecoline and its thick fibres, can cause OSF.^20^ In addition, arecoline has been shown to have exert cytotoxicity and genotoxicity, which not only causes DNA damage and oxidative stress, but also induces apoptosis of epithelial and vascular endothelial cells.^21^ The active ingredients in the areca nut, such as arecoline and flavonoids, enter into the submucosal tissue, while the autologous tissue changes into autoantigen, a phenomenon that causes an autoimmune reaction that leads to infiltration of inflammatory cells including activated T cells and macrophages.^22^ Studies have also shown that macrophages play a key role in fibrosis in the heart, lung, and kidney tissues, which occurs through inflammatory responses and overactivated damage repair.^23^, ^24^, ^25^

In this study, we explored the possible mechanism underlying IL‐13's action as an ‘inducing factor’ during stimulation of polarization of M2‐type macrophages, and its role in pathogenesis of fibrosis. Further, we investigated the effect of arecoline, the main pathogenic factor of OSF, on the function of FBs.

## 2 MATERIALS AND METHODS

### 2.1 Patients recruitment and selection criteria

The research protocol used in the current study was approved by the Institutional Review Board of Hunan Xiangya Stomatological Hospital, Central South University (approval no. 20220031). FBs were extracted from normal tissue from patients who met the following conditions: had normal local mucosa; no inflammation; no history of chewing areca nut; no medication history in the past 3 months; and no family history of OSF disease. Patients were stratified into the following subgroup, based on presentations: restricted mouth opening, white mucosa, palpable striae, firm texture, and OSF diagnosed via biopsy, and this part of the OSF tissue was used for subsequent experiments. A total of 85 tissue specimens, including 75 OSF and 10 normal tissues, were collected from patients at Hunan Xiangya Stomatological Hospital Central South University between 2018 and December 2021. The specimens had been previously diagnosed via pathological biopsy, with OSF histopathology confirmed by an expert pathologist.

### 2.2 Bioinformatics analysis

GSE64216, a dataset with mRNA expression profiles for OSF alongside normal samples, was downloaded from the Gene Expression Omnibus (GEO) database. Analysis of differentially expressed genes in the dataset was conducted at the following threshold: adjusted *p* (adj. *p*) <0.05, and log2 fold change (FC) >1.

### 2.3 Immunohistochemistry (IHC)

Paraffin‐embedded blocks were used to prepare OSF specimens, and 4‐μm sections were cut from them. These sections were then subjected to IHC by incubating them with specific primary antibodies at 4°C overnight: anti‐IL13 (Abcam, 1:500, Cambridge, MA, USA), anti‐CD163 (Abcam, 1:500), anti‐CD206 (Abcam, 1:1000), anti‐CD209 (Abcam, 1:500), (CD163/206/209 are the markers of M2 macrophages). The sections were then incubated with secondary antibodies using the Immunohistochemistry detection system kit (Bioss), dehydrated and the slides sealed with neutral resin. The sections were visualized under a microscope, where positive particles were stained brown. Optical density sum (IOD SUM), and area were measured using ImagePro Plus, and the mean density was calculated as follows: mean density = IOD SUM/area.

### 2.4 Quantitative reverse transcription polymerase chain reaction (qRT‐PCR)

OSF lesion tissues were collected from patients and subjected to RNA TRIzol Reagent according to the manufacturer's instructions. The RNA was quantified then converted to cDNA using the HiScriptIIQ RT SuperMix Kit (Nanjing Vazyme Biotech Co., Ltd). The cDNA was subjected to qRT‐ PCR, performed using the ChamQ Universal SYBR qPCR Master Mix (Vazyme), targeting IL13 gene (IL13‐F: 5’‐TAGCCGACCTCAGCCTT‐3′; and IL13‐R: 5’‐TGCCTGTGTGTGAAGTGG‐3′).

In this test, GAPDH (GAPDH‐F: 5’‐ACAGCCTCAAGATCATCAGC‐3′;

GAPDH‐R: 5’‐GGTCATGAGTCCTTCCACGAT‐3′) was included as the internal amplification control. Relative gene expression was calculated using the 2^−ΔΔCt^ method.

### 2.5 Isolation and culture of primary FBs cells

Experimental specimens were obtained from healthy mucosal tissues, resected during oral and maxillofacial surgery. The tissues were first washed with PBS supplemented with 20% penicillin and streptomycin, chopped into small pieces then centrifuged at 1000 rpm for 5 min. The tissue was digested with a mixture of Collagenase I (6 mg/mL) and Dispase (8 mg/mL) for 15 min, the cells counted, and transferred to a Petri dish. Culture medium was changed after every 3 days. FBs were cultured in Dulbecco‘s modified Eagle‘s medium (DMEM, Sigma), supplemented with 10% fetal bovine serum and 1% antibiotics, namely penicillin and streptomycin (100 μg/mL for each; Gibco). Cells at the 3rd to 6th passage three were used in subsequent experiments.

### 2.6 Immunofluorescence

Fourth‐generation FBs were seeded into 6‐well plates for immunocytochemical staining. Briefly, the cells were fixed with 4% paraformaldehyde, permeabilized with 0.3% Triton‐x‐100 at room temperature for 20 min, then blocked with 3% BSA at room temperature for 30 min. Next, they were incubated overnight with primary antibody (anti‐Vimentin, Proteintech) at 4 C, washed, then incubated for 1 h with diluted fluorescent secondary antibody (Goat anti‐Rabbit, Bioss) in a wet box. The sections were stained with DAPI for 5 min in the dark, and then incubated with the anti‐fluorescence quenching reagent. The sections were finally dried in the dark, then visualized under an inverted fluorescence microscope.

### 2.7 Cell proliferation assay

Proliferative activity of the FBs was assessed using the Cell Counting Assay Kit (CCK)‐8 (Dojindo Molecular Technologies), according to the manufacturer's instructions. Briefly, fourth‐generation cells were seeded into a 96‐well plate (1 × 10^4^ cells per well), and treated with different arecoline concentrations (0, 5, 10, 20, 30 and 40 μg/mL) (Sorabio). Cell growth was monitored using a Microplate Reader System (Rayto RT‐6100) at 0, 1, 2, 3 and 4 day after treatment.

### 2.8 ELISA

Levels of IL‐13 were quantified in the supernatant from FBs treated with arecoline using the ELISA kit (Zci Bio), following the manufacturer's instructions. Absorbance was measured at 450 nm wavelength using a microplate reader.

### 2.9 Extraction and maturation of macrophages

Cubital venous blood was collected from normal people in an EDTA anticoagulation tube, and peripheral blood mononuclear cells (PBMC) obtained via density gradient centrifugation and cultured in Roswell Park Memorial Institute‐1640 (RPMI‐1640) medium (containing 10% FBS). The next day, the supernatant was discarded and anchorage‐dependent cells were collected. The resulting monocytes were incubated with M‐CSF (PeproTech) for 7 days to obtain differentiated mature macrophages (M0‐phenotype).

### 2.10 Flow cytometry

The supernatant of FBs stimulated by arecoline was used as the conditioned medium for the experimental group whereas the supernatant of FBs without arecoline stimulation was utilized as the conditioned medium for the control group. After 48 h of treatment, the samples were analysed by flow cytometry. Cell density was adjusted to 1 × 10^7^ cells/mL, then macrophages incubated with FITC anti‐human CD14, PE anti‐human CD68, APC anti‐human CD209, PE anti‐human CD163, Cy5 anti‐human CD206 (BioLegend). Finally, the cells were detected with flow cytometry (Cyto‐Flex Beckman).

### 2.11 Statistical analysis

All experiments were conducted in triplicates. All statistical analyses were performed using SPSS software (version 25.0), and data were presented as means ± SD. Comparisons between two groups were achieved using the *t*‐test, whereas those among multiple groups were performed using one‐way anova or two‐way anova, followed by Tukey's post hoc test for mean separations. *p* < 0.05 was considered statistically significant. Figures were generated using Origin version 8.0.

## 3 RESULTS

### 3.1 M2 macrophages are abundant in OSF tissue

**FIGURE 1:**
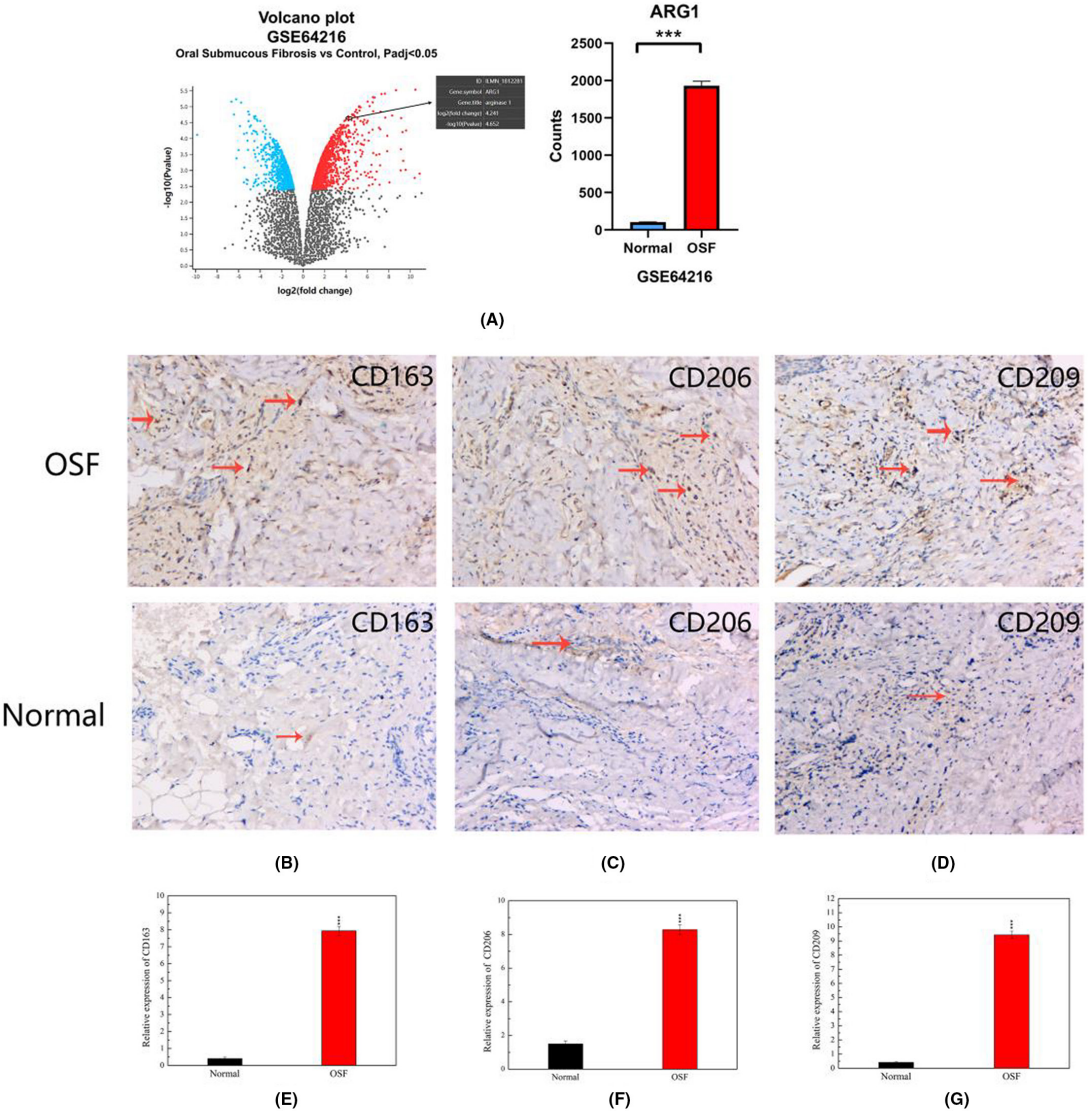
M2 macrophages was up‐regulated in OSF tissue (x100). (A) Volcano plot and histogram showed that the expression of ARG1 was significantly overexpressed in OSF tissues. (B‐D)Abundant expression of CD163, CD206, CD209 in human OSF as compared with normal mucosa. (E‐G) Quantitative analysis of ARG1 in human OSF and normal mucosa, ***p<0.001.

Bioinformatics analysis results revealed ARG1 was significantly overexpressed in OSF tissues. (Figure 1A). ARG1 is a specific marker of M2‐macrophages, and thus we hypothesized that M2‐macrophages are highly expressed in OSF tissues. Similarly, immunohistochemistry results corroborated the hypothesis, as evidenced by the higher abundance of M2‐macrophages in OSF relative to normal tissues (Figure 1B‐G).

### 3.2 IL‐13 is up‐regulated in OSF tissues

**FIGURE 2:**
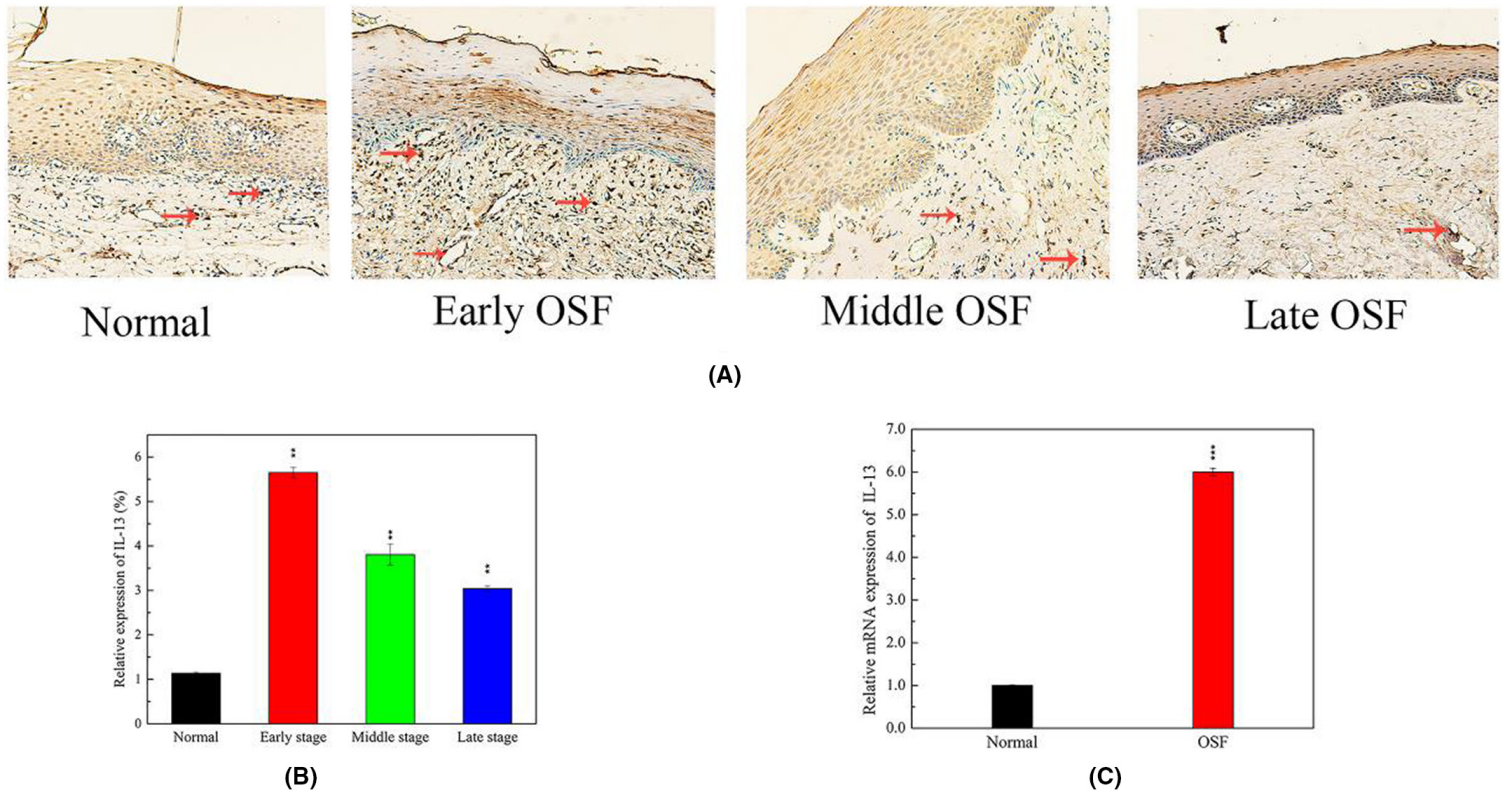
IL‐13 was up‐regulated in OSF tissue (x100) (A) IHC analysis of IL‐13 expression levels. Abundant expression of IL‐13 in human different stages of OSF as compared with normal mucosa. (B) Quantitative analysis of IL‐13 expression in OSF at different stages by ImagePro Plus software. (C) Quantitative real‐time PCR evaluated the relative IL‐13 mRNA levels in 25 OSF tissues and 15 normal tissues, **p < 0.01.

Next, we performed immunohistochemistry staining on normal (*n* = 10 cases) and OSF tissues at different stages (25 cases each at the early, metaphasic, and advanced stages), and found that IL‐13 was significantly upregulated in OSF relative to normal tissue across the stages (Figure 2A,B). Analysis of IL‐13 expression in epithelial, and vascular endothelial cells, as well as FBs revealed higher cell numbers and the highest IL‐13 expression in early OSF. In advanced OSF, the fibrotic tissue increased while the number of cells decreased, and these phenomena that were accompanied by low IL‐13 expression, although it was still higher than that in normal mucosal tissue. qRT‐PCR results showed that IL‐13 was upregulated in OSF, relative to normal tissues (Figure 2C).

### 3.3 Profiles of primary FBs

**FIGURE 3:**
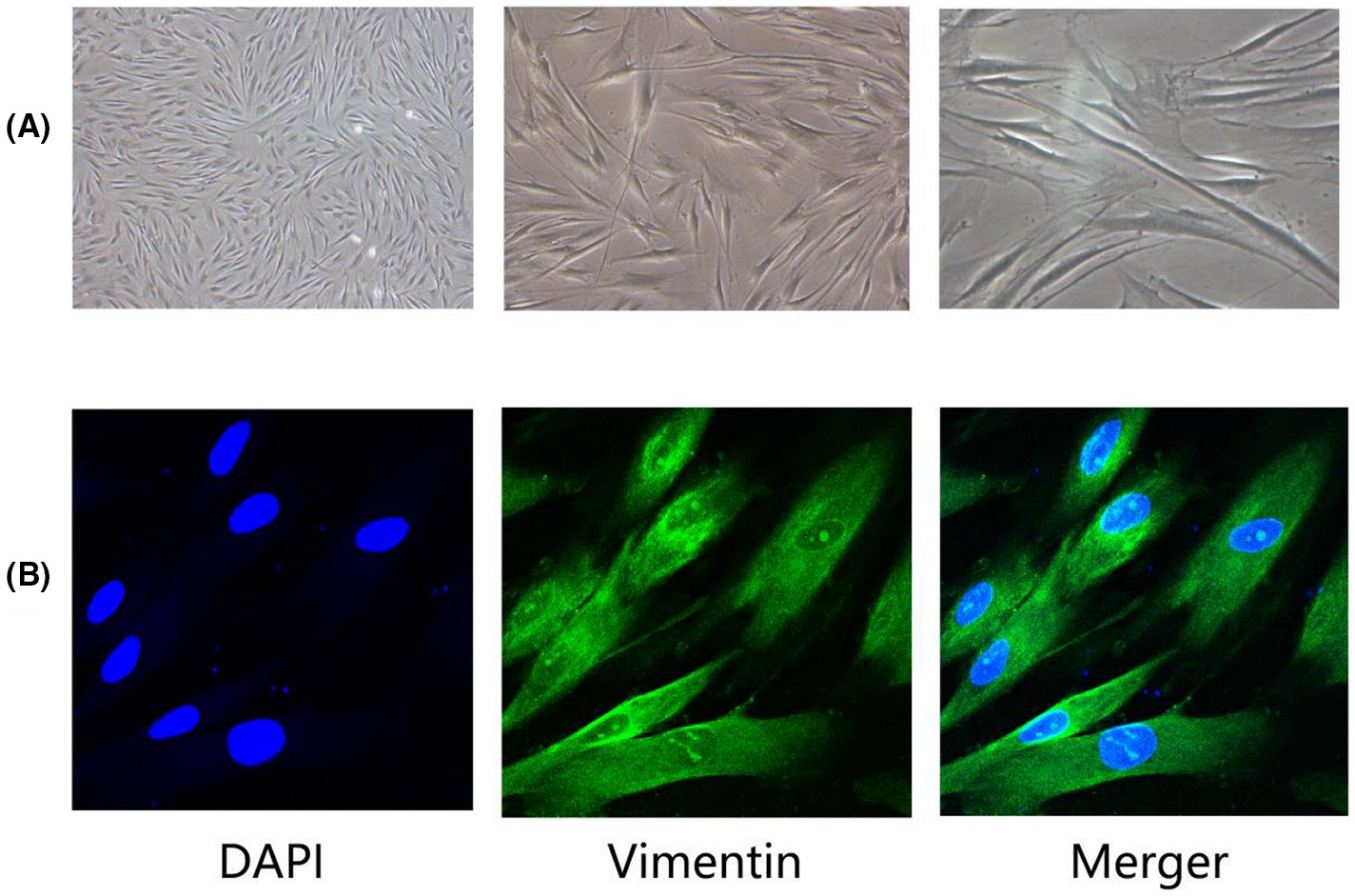
Extraction, culture, and identification of primary FBs (A) Optical microscope (x40, x100, x200): The cells were spindle‐shaped or irregularly triangular, with an oval nucleus in the center, cytoplasmic protrusions, and radial growth when growing. (B) Immunofluorescence identification of Vimentin protein staining in FBs. Under the microscope, Vimentin protein was mainly expressed in the cytoplasm of cells, blue was nuclear staining, green was cytoplasmic staining and represented Vimentin protein. (x200).

The isolated cells were either spindle‐shaped or irregularly triangular, with an oval nucleus in the center, as well as cytoplasmic protrusions and radial growth during growth (Figure 3A). The immunofluorescence staining results indicated the presence of a cytoplasmic anti‐vimentin antibody, which was later confirmed to be FBs (Figure 3B).

### 3.4 Arecoline treatment promotes IL‐13 secretion by FBs

**FIGURE 4:**
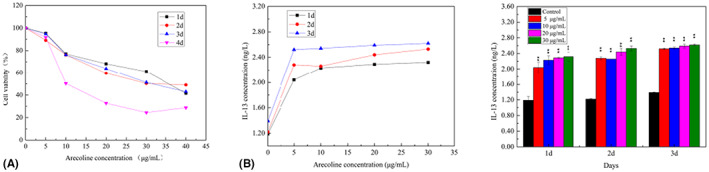
IL‐13 secreted by FBs was increased under the stimulation of arecoline (A) Arecoline is cytotoxic to FBs and can affect the cell viability of FBs. FBs has a dose and time dependence on arecoline, that is, with the increase in arecoline concentration and time, the proliferation of FBs gradually decreased. (B) With the changes in arecoline concentration (0, 5, 10, 20, 30 μg/mL) and culture time (0, 1, 2, 3days), the amount of IL‐13 produced by FBs showed increasing trend. At the concentration of 30 μg/mL, the most IL‐13 was produced at the 3 day of intervention. **p < 0.01.

To verify the cytotoxic effect of arecoline on FBs, we treated FBs with different arecoline concentrations over time, and compared with the control group. The IC50 of arecoline on FBs was 33.25 μg/mL after 3 days of treatment. Therefore, we selected 0‐30 μg/mL, 0–3 days as the arecoline concentration for use in subsequent experiments. Results indicated that IL‐13 was upregulated 1–3 days with increase in arecoline concentration (Figure 4B,C). We then selected 30 μg/mL arecoline for subsequent cell cultures and found that this concentration induced the highest IL‐13 secretion after 3 days of intervention. The supernatant at this time was used as the conditioned medium.

### 3.5 Differentiation of monocytes into mature M0 and their polarization into M2‐phenotypes

**FIGURE 5:**
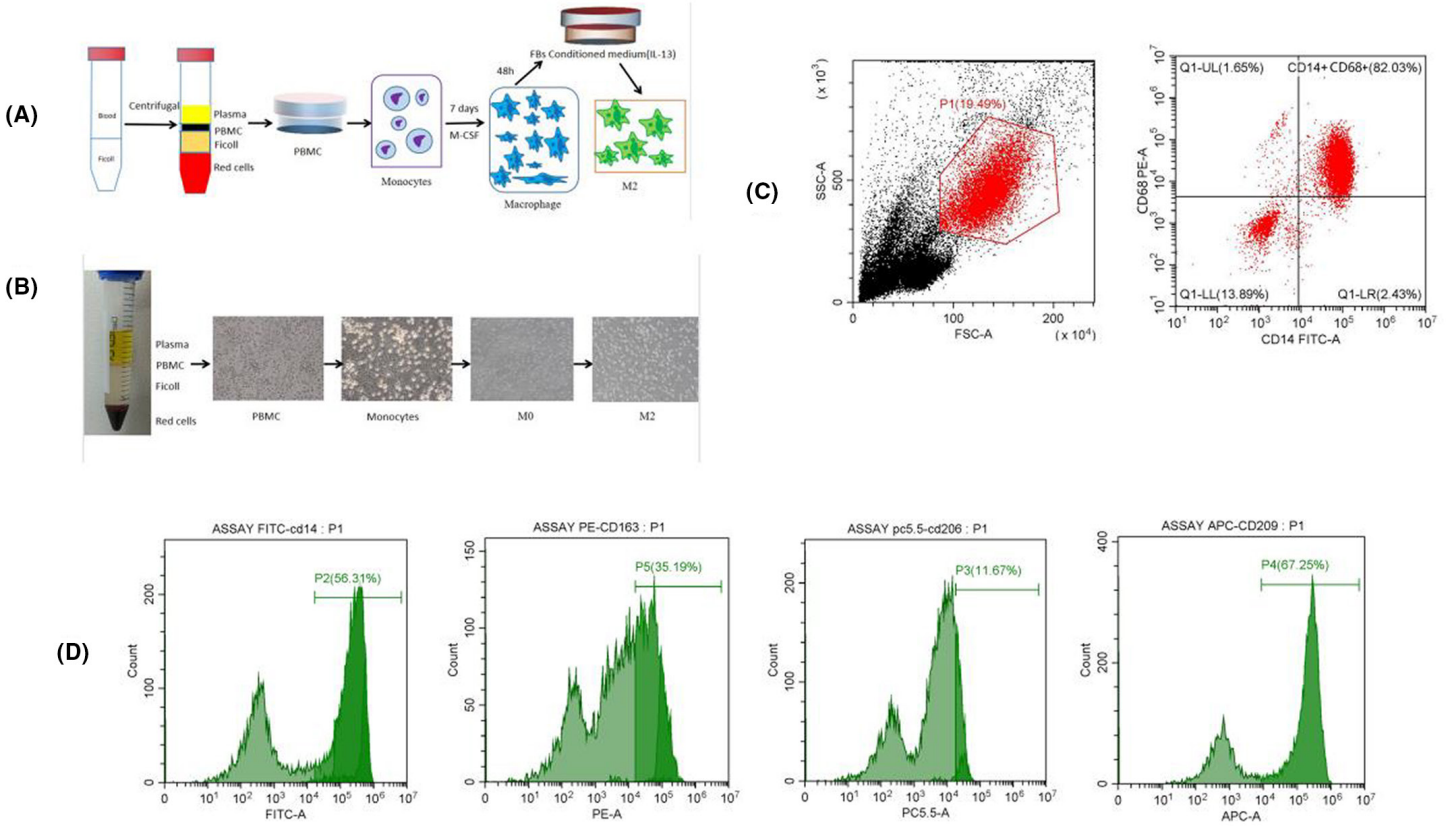
Differentiation of monocytes into mature M0‐macrophages and M0‐macrophage polarization into M2‐phenotypes (A) Schematic diagram of extracting macrophages. (B) The process of extracting macrophages was recorded under the microscope. Incubate monocytes with M‐CSF‐containing medium for 7 days to differentiate into macrophages (M0‐macrophages). The M0‐macrophages were then polarized for additional 2 days into conditioned medium,(X40). (C) Flow cytometry detected the extracted M0‐macrophage surface marker CD14^+^CD68^+^, which indicated that the extraction of macrophages was successful. (D) Detection of the extracted M2‐macrophage surface marker CD14^+^CD163^+^CD206^+^CD209^+^ by flow cytometry.

Fractions of granulocytes and mononuclear cells were first isolated from human peripheral blood, centrifuged and the supernatant discarded to obtain anchorage‐dependent cells (monocytes). Next, we cultured the monocytes in M‐CSF‐containing medium for 7 days to obtain target cells (M0 macrophages). Mature M0‐macrophages were then polarized into M2‐macrophages by induction with conditioned medium (Figure 5A,B). Flow cytometry results showed that our method was effective in extracting macrophages (Figure 5C,D). These results were validated via flow cytometric analysis of cell markers.

### 3.6 Conditioned media promotes M2 phenotype polarization of macrophages

**FIGURE 6:**
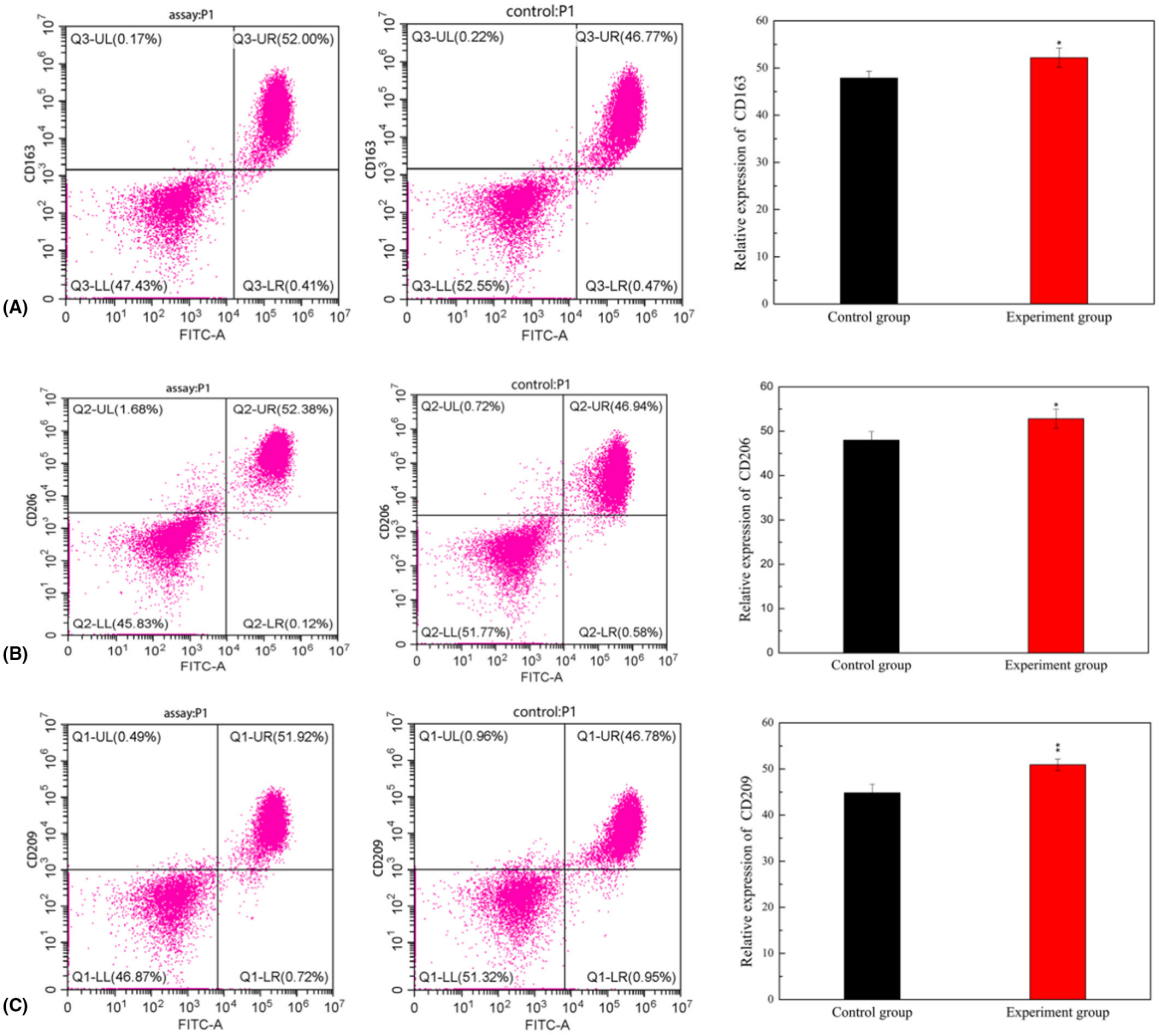
Conditioned media can promote M2 phenotype polarization of macrophages The proportion of M2‐macrophages were analysed with flow cytometry. (A‐C) shows the experimental group and control group of CD163^+^, CD206^+^, CD209^+^, respectively. The x‐coordinate was CD14^+^ cells, the y‐coordinate was CD163^+^, CD206^+^, CD209^+^ cells, and the upper right value was CD163^+^, CD206^+^, CD209^+^ proportion, *p < 0.05, **p < 0.01.

To investigate whether IL‐13 secreted by FBs under arecoline treatment can induce changes in polarization of macrophages, we first stimulated FBs with arecoline for 3 days, centrifuged the cells to obtain a supernatant for use as a conditioned medium. Next, we co‐cultured this medium with M0‐macrophages, with supernatant without arecoline used as a control group. Results revealed a slightly higher proportion of CD163 cells in the experimental (52.00%) than in the control group (46.77%). Similarly, more CD206 cells were recorded in the experimental (52.38%) than control group (46.94%). A similar trend was also observed with regard to CD209 cells, 51.92% and 46.78% in the experimental and control groups, respectively (Figure 6A). These differences were statistically significant (Figure 6B). Collectively, these results showed that arecoline promoted IL‐13 secretion by FBs which could subsequently cause macrophages to undergo M2‐phenotype polarization.

## 4 DISCUSION

The Results of the present study revealed that M2‐macrophages and IL‐13 were overexpressed in human OSF specimens relative to normal tissues. Notably, arecoline causes FBs to produce IL‐13, which subsequently induces polarization of M0‐macrophages into M2‐macrophages, thereby affecting occurrence and development of OSF. Macrophages are immune cells with multiple functions. For example, they are not only important targets for studying cellular phagocytosis, cellular immunity and molecular immunology, but are also involved in tissue inflammation and post‐repair.^26^ Notably, macrophages can be divided into pro‐inflammatory M1 or anti‐inflammatory M2‐phenotypes, based on different activation states and functions. M1‐macrophages promote inflammation by secreting pro‐inflammatory cytokines and chemokines, while M2‐macrophages promote tissue repair by inducing production of the fibrous tissue.^27^ Studies have shown that M2‐macrophages not only highly express mannose receptors Arginase‐1 (ARG1), CD163, CD206, CD209, but also play an important role in development of organ fibrosis. For instance, Li et al.^28^ found that induction of M2‐macrophage polarization could promote cardiac fibrosis in mice, while Wu et al.^29^ demonstrated that reduced M2‐macrophage polarization attenuates ccl4‐induced liver fibrosis in mice. In another study, Xie et al.^30^ demonstrated that pharmacological targeting of macrophage phenotype via the gut‐kidney axis reduced M2‐macrophages ameliorates renal fibrosis in mice. M2‐macrophages have also been shown to inhibit the antifibrotic function,^31^ which is consistent with our results. Collectively, these studies demonstrate that M2‐macrophages play a crucial promoting role in fibrosis. The Results from the present study showed that M2‐macrophages were highly expressed in OSF, which is consistent with the above studies. However, the cause of the observed polarization of M2‐macrophages in OSF is not known, necessitating further research.

The IL‐13 cytokine is an important factor in polarization of M2 macrophages. Based on our results, we speculate that the high expression of M2‐macrophages in OSF is due to upregulation of IL‐13 cytokines. Previous studies have reported that IL‐13 is highly expressed in lungs of patients with progressive fibrosing interstitial lung diseases.^13^, ^32^ Numerous studies have demonstrated IL‐13's involvement in fibrosis, suggesting that it may be a target for treatment of fibrotic diseases in the future. Functionally, the IL‐13 signalling pathway can activate FBs to produce extracellular matrix (ECM) and other constituent factors, which are required for collagen fibrogenesis.^33^ Results from the present study showed that IL‐13 was upregulated in FBs following arecoline intervention, which is consistent with previous studies. IL‐13 expression is higher in OSF than in normal tissues, but its expression level is higher in the early OSF than in the middle and advanced stage. The reasons for these findings may be as follows: IL‐13 is expressed in vascular endothelial cells, epithelial cells, immune cells, among other cells. Early OSF has pronounced edema, vascular dilatation and congestion, a large number of inflammatory cell infiltration, and high expression of IL‐13. However, in advanced stage, epithelial atrophy, vascular stenosis or occlusion, and collagen are increased whereas IL‐13 expression shows a decreasing trend.

At present, most scholars believe that arecoline is the main cause of OSF. Arecoline stimulates the oral mucosa to induce secretion of numerous cytokines, ECM and collagen, thereby resulting in excessive collagen synthesis and reduced degradation. This imbalance is caused by fibrous key conditions for the transformation.^34^ Furthermore, FBs are involved in degradation and synthesis of collagen, thus they play a key role in fibrosis formation. Our results showed that arecoline at a concentration of 5 μg/mL was cytotoxic to FBs, as evidenced by time‐ and dose‐dependent inhibitory effects. Increasing the concentration and duration of action resulted in continued upregulation of IL‐13 production by FBs. To avoid the influence on the subsequent experiments, we selected 0‐30 μg/mL as the experimental concentration, and found that IL‐13 cytokine level was highest at 30 μg/mL.

Next, we investigated whether IL‐13 secreted by FBs would induce changes to polarization of macrophages under the arecoline. To this end, we stimulated FBs with arecoline, then co‐cultured the supernatant (at this time considered conditioned medium), with M0‐macrophages. Results revealed that CD163, CD206 and CD209 were all upregulated, which is consistent with previous studies and is in line with our conjecture at the time.

In summary, we investigated the mechanism underlying the role of IL‐13 in M2‐macrophages and OSF progression. Our results demonstrated that arecoline can regulate IL‐13 cytokines produced by FBs and activates the transformation of polarized M0‐macrophages into M2‐macrophages. This result show that IL‐13 may be involved in the OSF pathogenesis.

## AUTHOR CONTRIBUTIONS

**Junhui Huang:** Conceptualization (lead); data curation (lead); investigation (lead); project administration (lead); resources (lead); validation (lead); writing – original draft (lead); writing – review and editing (lead). **Liping Wang:** Conceptualization (lead); data curation (lead); formal analysis (lead); funding acquisition (equal); investigation (lead); methodology (lead); resources (lead); software (lead); supervision (lead); validation (equal); writing – original draft (lead); writing – review and editing (lead). **Zhangui Tang:** Funding acquisition (lead); resources (equal); supervision (lead).

## CONFLICT OF INTEREST STATEMENT

The authors report no conflicts of interest.

## Data Availability

Research data are not shared.
